# Supplementary material for: Alcohol Consumption, Drinking Patterns, and Cognitive Performance in Young Adults: A Cross-Sectional and Longitudinal Analysis
Source: Nutrients. 2020 Jan 13;12(1):200. doi: 10.3390/nu12010200 (PMC7019481; doi:10.3390/nu12010200)
Supplement: Supplementary file 1 [file nutrients-12-00200-s001.pdf]

Supplementary A: Baseline questions for alcohol consumption and answer options

Below the baseline questions for alcohol consumption and answer options are listed. To compute an average value for alcohol consumption in grams per day, for each question the textual answers were recoded into numeric values, which was based on expert opinion.

Question 1: In the past year, how often did you drink?	Recoded Value
Never	0/30
Monthly	1/30
Several times per month	3/30
Weekly	5/30
Several times per week	12/30
Daily	30/30

Question 2: In the past year, how much did you drink, when drinking on a weekday (Monday, Tuesday, Wednesday, Thursday)

Question 3: In the past year, how much did you drink, when drinking on a weekend day (Friday, Saturday, Sunday)

Answer options for Question 2 and 3, including their recoded scoring:

□	1 or 2 glasses	1.5
□	3 or 4 glasses	3.5
□	5 or 6 glasses	5.5
□	7 or 9 glasses	8
□	10 or more glasses	12

Final calculation for alcohol consumption (gr / day): ((Q2 * 4 + Q3 *3)/7) * Q1

Supplementary B: Description and visualization of each cognitive task

Cognitive tasks

1 Odd one out (deductive reasoning task)

A 3* 3 grid of cells is displayed on the screen. Each cell contains a variable number of copies of a colored shape. The features that make up the objects in each cell (color, shape, number of copies) are related to each other according to a set of rules. The participant must deduce the rules that relate the object features and select the one cell whose contents do not correspond to those rules. To gain maximum points, the participant must solve as many problems as possible within 90 seconds. If the response is correct, the total score increases by one point and the next problem is more complex. If the response is incorrect, the total score decreases by 1 point.

Visualization:

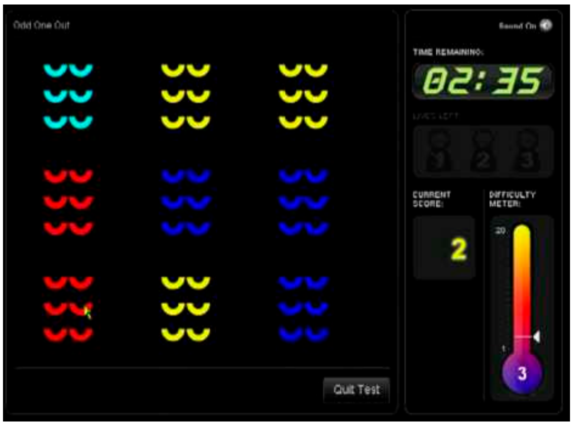

2 Spatial span task (working memory)

Spatial Span is a tool for measuring spatial short-term memory capacity. 16 squares are displayed in a 4 * 4 grid. A sub-set of the squares flash in a random sequence at a rate of 1 flash every 900 ms. Subsequently, the mouse cursor is displayed and a tone cues the participant to repeat the sequence by clicking on the squares in the same order in which they flashed. The test starts with four flashes and difficulty on subsequent trials is dynamically varied. If the participant responds correctly, the length of the next sequence increases by one flash, otherwise the length of the next sequence is one flash shorter. The test finishes after 3 errors.

Visualization:

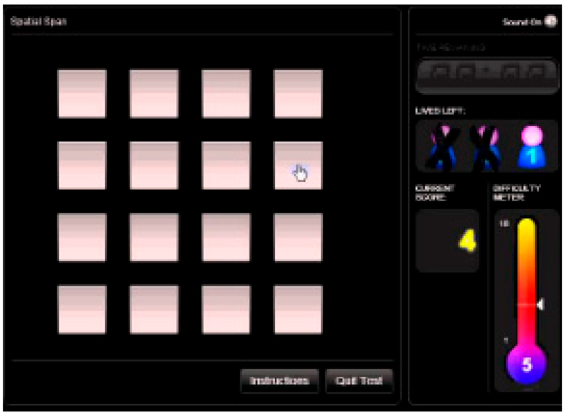

3 Spatial rotation task (concentration)

In this task two grids of colored squares are displayed to either side of the screen with one of the grids rotated by a multiple of 90 degrees. When rotated, the grids are either identical or differ by the position of just one square. In order to gain maximum points, the participant must indicate whether the grids are identical (clicking "match" or "mismatch"), solving as many problems as possible within 90 seconds. If the response is correct, the total score increases by the number of squares in the grid and subsequent trials have more squares. If the response is incorrect the total score decreases by the number of squares in the grid and subsequent trials have fewer squares. The first grids contain 4 colored squares each.

Visualization:

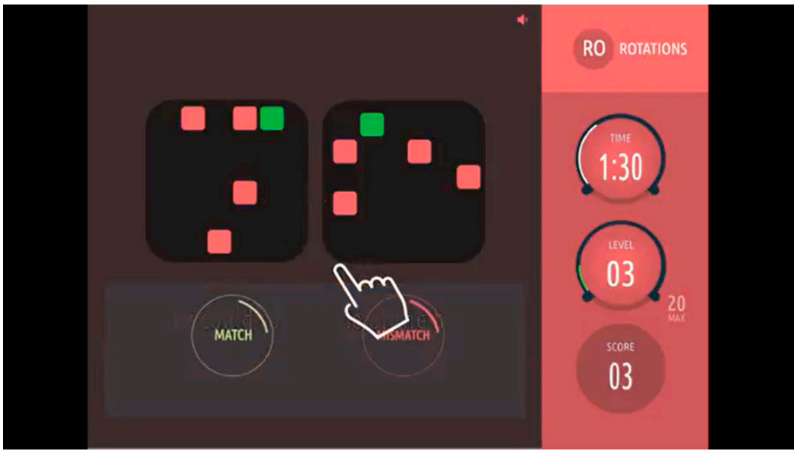

4 Digit span (working memory)

Participants view a sequence of digits that appear on the screen one after another. Subsequently, they repeat the sequence of numbers by entering them on the keyboard. Difficulty is dynamically varied with the number of digits to remember increasing or decreasing by 1 depending on whether the participant got the previous trial correct. The test ends after 3 errors.

Visualization:

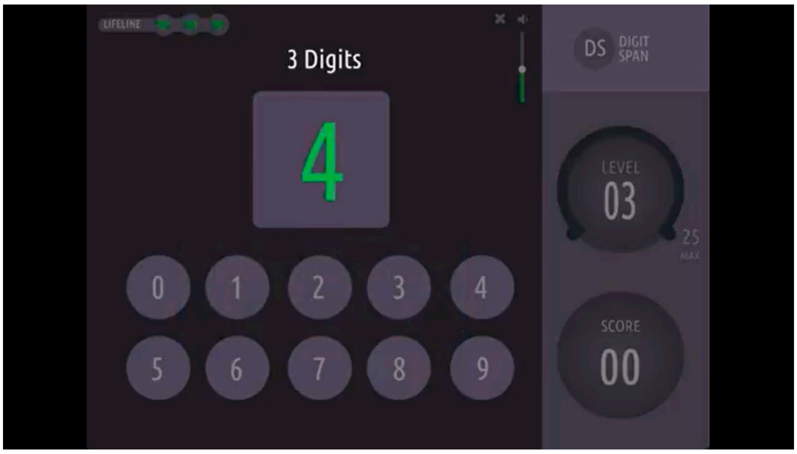

5 Paired association task (associate-learning)

Boxes are displayed at random locations on an invisible 5*5 grid. The boxes open one after another to reveal an enclosed object. Subsequently, the objects are displayed in random order in the center of the grid and the participant must click on the boxes that contained them. If the participant remembers all of the object-location paired associates correctly then the difficulty level on the subsequent trial increases by 1 object-box pair, otherwise it decreases by 1. After three errors the test ends. The test starts with just two boxes.

Visualization:

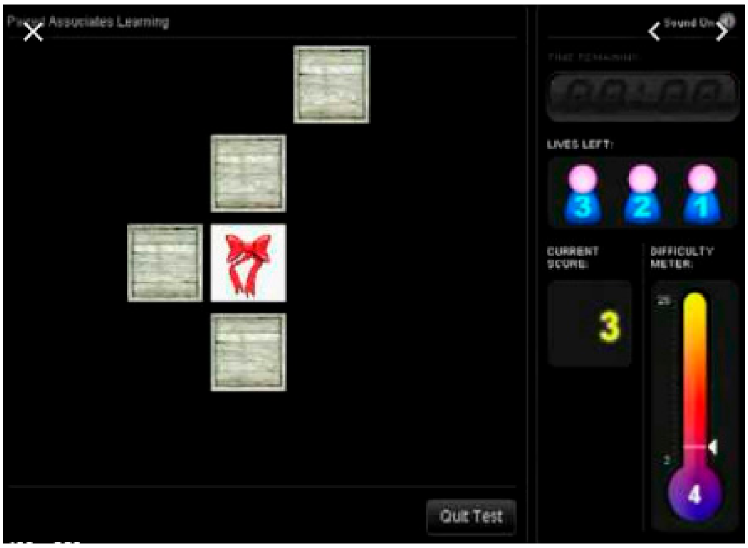

6 Tree task (spatial planning task)

This task is based on the Tower of London Task. The participant repositions the beads so that they are configured in ascending numerical order running from left to right and top to bottom of the tree. To gain maximum points, the participant must solve as many problems as possible in as few moves as possible within 3 minutes. Problems become progressively harder with the total number of moves required and the planning complexity increasing in steps. Trials are aborted if the participant makes more than twice the number of moves required to solve the problem. After each trial, the total score is incremented by adding the minimum number of moves required * 2 – the number of moves actually made, thereby rewarding efficient planning. The first problem can be solved in just 3 moves.

Visualization:

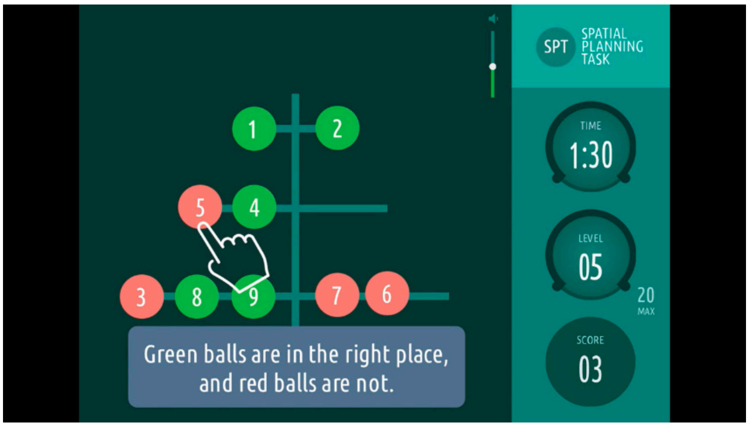

Supplementary C: Table of test statistics for alcohol consumption using baseline questionnaires vs EMA

	Means for alcohol consumption (gram per day)			
	Baseline questionnaire	Ecological momentary assessment	t	p
Week day	31	39	-6.22	< 0.001
Weekend day	40	45	-3.16	0.002

Table of test statistics for alcohol consumption and cognition

	Cognitive performance at baseline (F, p)						Cognitive performance at 1-year follow up (F, p)					
	OOO	SPT	SR	DS	PAT	TT	OOO	SPT	SR	DS	PAT	TT
Alcohol[a]	3.40, 0.07	6.36, 0.01	3.39, 0.07	0.01, 0.93	0.02, 0.90	0.61, 0.44	1.47, 0.23	0.64, 0.43	0.09, 0.77	0.23, 0.63	0.12, 0.73	<0.01, 0.93
Gender	0.68, 0.41	1.86, 0.17	0.02, 0.89	1.16, 0.28	0.07, 0.80	1.04, 0.31	0.01, 0.92	0.08, 0.85	2.06, 0.15	0.79, 0.38	1.07, 0.30	0.13, 0.72
Education	12.48, < 0.001	5.99, 0.01	8.32, < 0.01	4.28, 0.04	2.66, 0.10	2.51, 0.11	15.60, < 0.001	2.60, 0.11	0.82, 0.37	4.65, 0.03	8.45, <0.01	3.14, 0.08
alcohol:gender	0.42, 0.52	0.05 0.82	0.48, 0.49	0.71, 0.40	1.28, 0.26	0.01, 0.94	1.53, 0.22	0.61, 0.43	0.35, 0.55	0.59, 0.44	0.30, 0.58	0.12, 0.73
alcohol:education	1.81, 0.18	0.57, 0.45	0.95, 0.33	0.05, 0.83	0.36, 0.55	0.02, 0.88	0.64, 0.43	< 0.01, 0.94	0.69, 0.41	0.53, 0.47	1.00, 0.32	< 0.01, 0.97
gender:education	< 0.01, 0.93	0.16, 0.69	0.78, 0.38	0.75, 0.39	0.13, 0.72	0.72, 0.40	5.55, 0.02	0.74, 0.40	2.06, 0.15	3.41, 0.07	0.08, 0.77	2.05, 0.15
Alcohol[b]	-	-	-	-	-	-	0.03, 0.86	3.41, 0.07	2.13, 0.14	0.13, 0.72	0.05, 0.82	0.17, 0.68
Gender	-	-	-	-	-	-	0.45, 0.50	< 0.001, 0.98	3.07, 0.08	0.42, 0.51	1.23, 0.27	0.24, 0.63
Education	-	-	-	-	-	-	16.46, < 0.001	2.19, 0.14	0.59, 0.45	5.00, 0.03	10.15, <0.01	3.01, 0.08
alcohol:gender	-	-	-	-	-	-	0.55, 0.46	3.71, 0.06	0.49, 0.48	0.23, 0.63	0.51, 0.48	1.17, 0.28
alcohol:education	-	-	-	-	-	-	0.19, 0.67	0.01, 0.91	0.40, 0.53	0.95, 0.33	3.66, 0.06	0.91, 0.34
gender:education	-	-	-	-	-	-	5.28, 0.02	0.42, 0.52	2.47, 0.12	2.70, 0.10	<0.001 0.99	1.55, 0.21
Alcohol drinking pattern[c]	1.82, 0.18	6.44, 0.01	0.001 , 0.96	0.44, 0.51	0.06, 0.81	0.11, 0.74	0.34, 0.56	2.82, 0.09	1.98, 0.16	0.04, 0.85	0.34, 0.56	0.05, 0.83
Gender	0.07, 0.79	0.42, 0.52	0.59, 0.44	1.10, 0.30	0.05, 0.82	1.44, 0.23	0.56, 0.45	0.21, 0.65	1.63, 0.20	0.52, 0.47	0.83, 0.36	0.11, 0.74
Education	12.67, <	5.91, 0.02	9.06, <	4.18, 0.04	2.65, 0.10	2.54, 0.11	17.1, <	1.94, 0.16	0.49, 0.49	5.00, 0.03	10.52, < 0.01	3.03, 0.08

	0.001		0.01				0.001					
alcohol:gender	0.71, 0.40	1.94, 0.16	2.04, 0.15	5.32, 0.02	3.36, 0.07	0.07, 0.78	2.22, 0.14	0.23, 0.63	< 0.01, 0.93	<0.01, 0.97	0.12, 0.73	1.19, 0.28
alcohol:education	1.99, 0.16	1.04, 0.31	0.80, 0.37	0.11, 0.74	0.05, 0.82	0.51, 0.47	1.68, 0.20	0.81, 0.37	0.20, 0.65	< .001, 0.99	0.32, 0.57	0.08, 0.78
gender:education	0.23, 0.63	0.09, 0.76	1.11, 0.29	0.37, 0.54	0.40, 0.53	0.73, 0.39	5.12, 0.02	0.46, 0.50	2.72, 0.10	3.38, 0.07	0.29, 0.59	2.37, 0.12
Alcohol drinking pattern[d]	-	-	-	-	-	-	0.90, 0.34	0.37, 0.54	< 0.001, 0.98	0.06, 0.81	0.05, 0.82	0.001, 0.97
Gender	-	-	-	-	-	-	0.43, 0.51	< 0.01, 0.96	1.53, 0.22	0.47, 0.50	0.75, 0.39	0.09, 0.77
Education	-	-	-	-	-	-	16.98, < 0.001	2.65, 0.10	0.92, 0.34	4.51, 0.03	8.59, < 0.01	3.08, 0.08
alcohol:gender	-	-	-	-	-	-	0.07, 0.79	0.76, 0.38	2.65, 0.10	1.01, 0.32	< 0.001, 0.99	0.41, 0.52
alcohol:education	-	-	-	-	-	-	0.09, 0.76	0.12, 0.72	1.52, 0.23	0.63, 0.43	0.12, 0.73	0.10, 0.75
gender:education	-	-	-	-	-	-	5.47, 0.020	0.58, 0.45	2.14, 0.14	3.29, 0.07	0.34 0.56	1.66, 0.20

OOO: Odd One Out; SPT; Spatial Planning Task; SR: Spatial Rotation; DS: Digit Span; PAT: Paired Associates Task; TT: Tree Task;

[a] Alcohol consumption as determined by four weeks of ecological momentary assessment at baseline;

[b] Alcohol consumption as determined by four weeks of ecological momentary assessment at 1-year follow up;

[c] Alcohol consumption pattern as determined by four weeks of ecological momentary assessment at baseline;

[d] Alcohol consumption pattern as determined by four weeks of ecological momentary assessment at 1-year follow up;
